# COVID and World Stock Markets: A Comprehensive Discussion

**DOI:** 10.3389/fpsyg.2021.763346

**Published:** 2022-02-28

**Authors:** Shaista Jabeen, Muhammad Farhan, Muhammad Ahmad Zaka, Muhammad Fiaz, Mobina Farasat

**Affiliations:** ^1^Department of Management Sciences, Lahore College for Women University, Lahore, Pakistan; ^2^Department of Management Sciences, National University of Modern Languages, Islamabad, Pakistan

**Keywords:** COVID-19, stock markets, market indices, behavioral finance, SARS

## Abstract

The COVID-19 outbreak has disturbed the victims' economic conditions and posed a significant threat to economies worldwide and their respective financial markets. The majority of the world stock markets have suffered losses in the trillions of dollars, and international financial institutions were forced to reduce their forecasted growth for 2020 and the years to come. The current research deals with the impact of the COVID-19 pandemic on the global stock markets. It has focused on the contingent effects of previous and current pandemics on the financial markets. It has also elaborated on the pandemic impact on diverse pillars of the economy. Irrespective of all these destructive effects of the pandemic, still hopes are there for a sharp rise and speedy improvement in global stock markets' performance.

## Introduction

The world is experiencing the worst health and economic disaster in the shape of COVID-19 pandemic. Dealing with this pandemic is the most challenging task being faced by human beings since the Second World War (Maqsood et al., 2021). Coronavirus has pushed the markets toward the danger zone. The market panic has been started. This disease is contagious even before it shows obvious symptoms. It is quite difficult to hold people in quarantine in this outbreak. That's the narrative, and we haven't gotten very far into it yet. So, the potential for market disruption because of a scary narrative is quite high.

—Robert James Shriller, Nobel Memorial Prize Winner in Economic Sciences, 2013.

The epidemiological perspectives are not required to be understood here. Currently, well-informed individuals ought to have some know-how about the basics of contagious diseases. Times of fear are also times of rumor and misinformation; knowledge is the antidote (Baldwin et al., 2020). The COVID-19 outbreak was officially reported in the Wuhan City of China in December 2019 and covered all continents of this globe other than Antarctica (Hui et al., 2020). COVID-19 is a distinctive black swan event, and we are unaware of its existence, expansion, breadth, depth, magnitude, and even its disappearance (He P. et al., 2020; He Q. et al., 2020). World Health Organization (WHO) officially declared COVID-19 a pandemic on 11th March 2020 (Cucinotta and Vanelli, 2020). The pandemic has severely hit global economies (Shafi et al., 2020). It has disrupted the life and lifestyle of almost everyone (Aqeel et al., 2021). Almost no one has been left untouched. Another pandemic of information and misinformation is keeping pace with it during this pandemic, spreading fear and anxiety (Koley and Dhole, 2020). The outbreak has changed the outlook of this globe within no time at all. Human beings are struggling with the long-lasting effects of this disease and the unforgettable reality of their existence which has never happened before. The pandemic affected more than 107 million people, with around 2.3 million causalities, and the numbers of cases are escalated day by day. The alarming point is the growth factor of this disease, where 100 contaminated cases create another 10,000 within a very limited time (Bagchi et al., 2020).

The people from this generation have seen wars. They have seen the collapse of the Soviet Union. They have seen extremely dangerous terrorist attacks. They have seen the burst of financial bubbles, and they have seen the effects of climate change. However, they had not seen anything like the coronavirus before. A similar case has not existed for more than one hundred years. They were not ready for it, and they did not know how to respond to it. Since it was something that no one had any prior experience with, the pandemic has also led to reconsidering some things which were always previously thought either right or wrong (Sharma, 2021).

The COVID-19 pandemic has spread globally, has made millions of people sick, and triggered an international response spearheaded by the World Health Organization to stop its spread. From Wuhan, China, it spread like wildfire. The virus has now visited almost every nation in the world, bringing helplessness and death with it. None are spared, and in some way or another, almost everyone has become a victim. In a recent message, the WHO warned that the worst is yet to come. The coronavirus has not only triggered disease and death, and it has affected almost every aspect human life. There is a long list of disruptions to daily life in the cities and states with lockdown, global sporting events, weddings, social events, post-poned ceremonies; all this has elicited the global crisis. Moreover, industries worldwide have been affected; stock markets have been reported in record downfall; airlines, travel, tourism, and hospitality sectors are the major victims of this pandemic. A significant disaster is job loss in various sectors (Koley and Dhole, 2020).

Crucial and groundbreaking strategies are required to protect not only human lives but also to safeguard economies and uplift economic growth and financial health. Nations are exposed to a global health crisis, the like of which has not occurred for a century. This crisis is killing human beings, enhancing human distress, and upsetting the lives of individuals. This can be considered a sort of human, social, and economic crisis (Mishra, 2020). The best efforts by governments from every country have failed to halt its spread: cities were put under lockdown; people were advised to stay at home; international borders were closed; travel bans at local, national and international level were imposed; markets, schools, universities and shopping complexes were closed. Quarantine and self-isolation have been advised to stop the spread of COVID-19. The virus has triggered an unprecedented global crisis which led the WHO to provide technical guidance for government authorities, healthcare workers, and other key stakeholders to respond to community spread (Koley and Dhole, 2020).

In the intermingled economies, the Covid-19 pandemic came as a global distress that affects both the demand and supply side concurrently. Rapidly growing infectivity limits labor supply and badly affects productivity, whereas supply disruptions are also caused by social distancing, lockdowns and industry closures. On the other hand, disruption on the demand side is caused by reduced consumption, unemployment, and income loss and these economic prospects result in reduced company investment. The unpredictability about the path, instance, enormity and impact of Covid-19 could create a vicious cycle of redundancy, less consumption, and business closures, leading to financial distress. To identify and determine this extraordinary shock is the key challenge for the experiential analysis of this pandemic. The unprecedented nature of COVID-19 makes it difficult to recognize its non-linear effects, cross-country spillovers, and quantify unobserved factors to compose forecasts (Chudik et al., 2020).

International institutions including the FAO, ILO, IFAD, and WHO jointly declared this pandemic a global challenge to food systems, public health, trade, and industry. Overwhelming social and economic disruptions put tens of millions of individuals at risk of falling below the poverty line. According to another approximation, by the end of this year, the number of undernourished people could increase by up to 32 million, which are ~690 million at present. It also poses an existential threat to a considerable number of business ventures. The world has a 3.3 billion workforce and ~50% of which are near to being unemployed. Significant individuals are informal workers with limited access to productive assets, quality health, and the majority lack social protection. Due to lockdowns, they lost their means to earn money and became incapable of feeding themselves and their families because for most their daily food depends upon their daily wages earned. Such a devastating effect on the entire food chain has exposed the vulnerability of this pandemic. Farmers have no access to markets, nor can buy inputs or sell their output and result in a reduced harvest. In addition to market shutdown, trade limitations, border closures, and detention measures dislocate food supply chains nationally and internationally, which badly influenced a healthy diet. Small scale farmers are the soft target of COVID-19 and placed nutrition and food security of the most marginalized population under threat, as income producers fall ill, die, or otherwise lose their work (Chriscaden, 2020).

It is still difficult to understand the recovery due to the development of vaccines. To understand corona's economic impact, the following charts and maps exhibit real statistics so far.

### Impact on Jobs

A report published by the OECD (2020) shows the impact of COVID-19 and containment measures on OECD economies where people were prohibited from going to work, resulting in a significant drop in business activity and extraordinary job losses. In some countries, millions have been moved to reduced hours and most people worked up to ten times fewer hours. Moreover, the rate of entire job loss is also very high. Some people are more exposed to this pandemic than others. As young people and women workers are at greater risk due to less secured and unskilled jobs. They are also associated with the industries most affected by this unprecedented shock, including restaurants, cafés and tourism.

### Causing Recession

Worldwide economic downturn caused by the COVID-19 pandemic forced the Organization for Economic Cooperation and Development (OECD), International Monetary Fund (IMF), and World Bank (WB) to revise their forecasts and reported a significant decline in the projected rate of growth in late 2019 and mid-2020. Such deterioration can be seen in the IMF figures in which global economic growth forecasts declined from +3.4% to −4.4% during October 2019 and October 2020. In the same way, OECD also revised its forecast and lowered the growth rate from positive 2.9% in November to −4.5% in September 2020. In June 2020, OECD anticipated the blow of another wave of infections.

### Impact on Travel

The travel industry is one of those acutely damaged industries due to lockdowns, border closures, and abandoned flight operations. Airlines are not only canceling flights, but customers also restrict themselves from holidays and business trips. A recently discovered subsequent wave of COVID-19 has forced national and international airlines to promulgate new travel restrictions and tighten their policies. While providing data of 2020, Flight tracking service Flight Radar 24 reveals a huge hit in number of flights worldwide and requires a long way to recover (Jones et al., 2021).

### Impact on Tourism

Tourism is another badly affected industry due to this unprecedented pandemic. The World Tourism Organization, also known as UNWTO (2020) marked this pandemic as a serious threat to the travel and tourism sector. Many jurisdictions put restrictions on international travel to restrict the spread of the virus; some fully closed their borders, resulting in a massive decline in demand. In 2020, tourism reported a loss of ~1 billion tourists, equivalent to US$ 1.1 trillion in international tourism receipts. This decline in international tourism could cause an ~$2 trillion loss in global GDP, over 2% of the global GDP in 2019. While predicting a rebound in the global tourism industry, UNWTO presented an extended scenario for the year 2021 to the year 2024. According to them, global tourism will start recovery from the second half of the year 2021 but it will take 2.5 to 4 years to return to 2019.

### Impact on Stock Markets

The capital markets are at the front line of any country's economy, and the stock markets are considered the indicator of any economy (He P. et al., 2020; He Q. et al., 2020). The COVID-19 outbreak has disturbed the victims' economic conditions and posed a significant threat to the worldwide economies and their respective financial markets (Barro et al., 2020; Ramelli and Wagner, 2020). The majority of the world stock markets have suffered in terms of trillion-dollar losses (Lyócsa et al., 2020) and international financial institutions were forced to reduce their forecasted growth for 2020 and the years to come (Boone et al., 2020). The root cause of this severe decline is the exposure of stock markets to several risks, for instance, the global financial crisis of 2008, which had pushed these markets in a melting position (Dang and Nguyen, 2020). The current pandemic has affected the global stock markets significantly compared to the SARS virus, which was spread in 2003 as China has got tremendous development in comparison to the last 17 years and recognized as a leading economy of the world and also a global production hub, manufacturing the highly demanded technology products (Alameer et al., 2019).

## Effects of Previous Pandemics on Stock Markets

Scholars have argued that previous pandemics triggered fragile stock markets (Chen et al., 2018) and impeded stock market participants' decision-making capacity by reducing their active involvement in stock market trading (Dong and Heo, 2014). The literature has provided empirical evidence of the stock market reactions to significant systematic events. The research has shown the cyclical nature of the stock market reactions and the factors that affected the stock markets (Keating, 2001). The historical performance of stock markets has been documented in the previous literature regarding influenza and other major epidemics. Similarly, the scholars have examined the influences of significant events on the stock markets, i.e., Severe Acute Respiratory Syndrome (SARS), (Chen et al., 2018), natural disasters (Caporale et al., 2019), corporate events (Ranju and Mallikarjunappa, 2019), public news, and political events (Bash and Alsaifi, 2019). Some other studies have also demonstrated that SARS in 2003 weakened the Taiwanese economy (Chen et al., 2007) and regional stock markets (Chen et al., 2018).

The previous studies have comprehensively examined the association between outbreaks and stock market performance. Kalra et al. (1993) investigated the disaster of the Soviet Chernobyl nuclear power plant. Delisle (2003) recognized that effects were of greater intensity after SARS (2003) than the Asian financial crisis. Nippani and Washer (2004) investigated the effects of SARS on global financial markets and found that it influenced the markets of China and Vietnam. Lee and and McKibbin (2004) reported the strong effect of SARS on human beings and financial integration. Loh (2006) explained a robust linkage between SARS and airline stocks performance in Canada, China, Hong Kong, Singapore, and Thailand and illustrated that the stocks of the aviation sector are more sensitive than non-aviation stocks. MckKibbin and Sidorenko (2006) investigated the influenza epidemic's impact on the global economy's growth by considering its diverse magnitudes like slight, moderate and intense. Moreover, Chen et al. (2007) noticed the negative effects of SARS on the hotel industry's stock prices in Taiwan. They also investigated the significant influence of SARS on the four major stock markets of Asia and China. Nikkinen et al. (2008) discovered the impact of the 9/11 incident on the global stock prices; however, the markets recovered rapidly. Al Rjoub (2009) also studied the influence of financial crisis on stock market.

Besides, Kaplanski and Levy (2010) studied the effect of aviation accidents on stock returns and established that price fluctuations are sensitive to such incidents. Al Rjoub (2011) and Al Rjoub and Azzam (2012) investigated the impact of the Mexican tequila crisis (1994), Asian-Russian financial crisis (1997–98), 9/11 incident, Iraq war (2004), financial crisis (2005), and global financial crisis (2008–09) on the stock compensation behavior in Jordan's Stock Exchange. Righi and Ceretta (2011) established the positive effect of the European debt crisis (2010) on European markets' risk aptitude, especially the German, French, and British markets. Schwert (2011) explored the variabilities in the prices of US stocks during the financial crisis. Mctier et al. (2011) found the negative impact of Flu on the intensity of trading activities and stock returns in the USA. Besides, Rengasamy (2012) examined the effect of Eurozone sovereign debt-related policy announcements, development rewards, and stock market volatility on Brazil, Russia, India, China, and South Africa. Karlsson and Nilsson (2014) found the negative impact of the 1918 Spanish flu epidemic on capital returns. Lanfear et al. (2018) conducted a study to explore the effect of cyclones on stock returns, and they observed the effect of emergencies on stock returns. Chen et al. (2018) examined the influence of SARS on Asian financial markets.

## Brief Overview of Literature

Studies have elaborated on the performance of global stock markets affecting the COVID-19 outbreak (Ahmar and del Val, 2020; Al-Awadhi et al., 2020; Liu et al., 2020; Zhang et al., 2020). The pandemic has decreased investors' confidence level in the stock market as the market uncertainty was very high (Liu et al., 2020). Iyke (2020) explained that COVID-19 has robust and continual negative effects on the global economy. Ahmar and del Val (2020) used ARIMA and SutteARIMA and forecasted the short-term impact of COVID-19 on Spain's IBEX index. They further explained that SutteARIMA is the better statistical measure in forecasting such impact.

Moreover, Alam et al. (2020) explained that pandemic has greatly hit Australia's capital market right from the start of 2020. The stock market has shown a bearish trend, though some sectors were at high risk and others have performed well. The researchers have focused on initial volatility and sectoral returns in eight different sectors. They have analyzed the data using the event study method and 10-days window for the official announcements of COVID-19 events in Australia. The findings revealed that some sectors performed well on the day of the announcement. Simultaneously, some others also showed good performance after the announcement except the transportation sector, which performed poorly.

The pandemic has posed severe challenges to the global economies (Wang et al., 2021) and it has also created mental health issues (Abbas et al., 2021). Chowdhury et al. (2020) examined the impact of COVID-19 on economic activities and stock markets worldwide. The study has targeted 12 countries from four continents from January-April 2020 by using the panel data. The stock market impact was measured using the event study method, and economic impact was measured using the panel vector autoregressive model. The results showed the extremely negative effects of pandemic variables on stock returns. Singh et al. (2020) investigated the influence of COVID-19 on the stock markets of G-20 states. The study used an event study for measuring abnormal returns and panel data to describe the causes of abnormal returns. The data consisted of 58 days of post-COVID period news provided by international media and 120 days before the event. The findings exhibited the significant negative abnormal returns during the event days. Liu et al. (2020) also examined the pandemic's effect on the most affected countries' stock markets by using the event study. The researchers revealed the negative effects of COVID-19 on the stock markets' performance.

He P. et al. (2020) and He Q. et al. (2020) also used the event study method to explore the impact of COVID-19 on Chinese industries and stock market performance. It has been observed that some industries were severely affected by the pandemic (mining, environment etc.). However, some other industries have faced limited effects of an outbreak (manufacturing, education etc.). Machmuddah et al. (2020) used the event study method to observe consumer goods' share prices before and after COVID-19. The data about daily stock prices and stock trade volume has been collected before and after the pandemic. Significant differences have been observed between daily closing prices and stock trade volume before and after the pandemic. Liu et al. (2020) used an event study method to study the short term impact of the outbreak on the stock market indices of 21 countries strongly affected by pandemic (Italy, UK, Germany etc.). Asian countries have taken the severe negative effect of the pandemic as compared to other states. Khatatbeh et al. (2020) also applied the event study method to discover the impact of COVID-19 on some targeted countries' stock indices by employing the daily stock prices and found a significant negative impact on returns.

Al-Awadhi et al. (2020) investigated the association of pandemic and stock market outcomes in the Chinese stock market. The findings showed the effect of pandemic cases and deaths on the stock returns of different organizations. Baker et al. (2020) claimed that COVID-19 strongly affects the US stock market compared to previous epidemics, including the Spanish Flu. Eighteen market jumps were observed from February-March 2020. The market jumps were considered to be the largest ones since 1990. The causes behind such jumps were the lockdowns and production cut. Ozili and Arun (2020) described that COVID-19 uncertainty and the fear of losing profit have resulted in 6 trillion USD in the global stock market on 24th February 2020. Similarly, the S&P 500 index has faced a loss of 5 trillion US dollar. The research also demonstrated the significant influence the pandemic on the opening, highest, and lowest stock indices in the US. Ngwakwe (2020) illustrated the influence of COVID-19 outbreak on some targeted stock indices (SSE, Euronext, and DJIA) by collecting the data for 50 days before and 50 days within the pandemic. The differential effects of the pandemic were observed in different stock markets. DJIA stock returns were decreased, SSE increased, however, S&P 500 index and Euronext 100 revealed insignificant effects.

He P. et al. (2020) and He Q. et al. (2020) examined the direct effects of COVID-19 spillovers on the stock market. The daily return data has collected from China, Italy, South Korea, France, Spain, Germany, Japan and the USA. The findings showed the negative short term effects of COVID-19 on the stock indices. Zhang et al. (2020) elaborated the impact of pandemic fear on the pattern of systematic risk and country-specific risk in the global financial markets. They explained the volatile nature of financial markets and the huge impact of uncertain market conditions on financial market risk. Sobieralski (2020) evaluated the effect of COVID-19 on employment and the aviation industry. The stock returns of China and US stocks have declined at a record level. Qin et al. (2020) investigated the influence of outbreak on oil markets.

Sansa (2020) explained the association between COVID-19 recorded cases and financial markets systems of SSE and DJIA during March 2020. Aslam et al. (2020) studied the impact of COVID-19 on 56 global stock market (developed, developing, emerging, and frontier) indices by using the network method. Topcu and Gulal (2020) have discovered a huge impact on Asian markets as compared to European markets. Ashraf (2020) explained that confirmed cases more strongly affect the stock market than deaths. Czech et al. (2020) used the TGARCH model and found the negative impact of COVID-19 on Visegrad stock market indices. They discovered that stock markets were seriously affected when the disease's nature was changed from epidemic to pandemic.

Zhang et al. (2020) also investigated the influence of COVID-19 on the stock markets of 10 countries. It was concluded that European stock markets showed connectivity during the outbreak; however, US markets could not show a leading role before and during the pandemic. Okorie and Lin (2021) discovered the occurrence of financial contagion during the pandemic. Corbet et al. (2020) presented some interesting insights. They illustrated that pandemic greatly affected the companies having names related to the virus, although these companies were not related to the virus.

## Methods

The current research work basically pertains to the comprehensive discussion about the past present and future of world stock markets. For the sake of achieving the research aims, it has also presented a somehow brief yet inclusive debate about the happenings in the renowned stock markets. It has focused on the major market indices belong to different regions and also it has attempted to explain the actual position of some famous indices with the help of underlying real time data based graphs. Its major contribution is presenting the diverse opinions of traditional and behavioral finance regarding the behavior of stock market participants.

## A General Debate About Stock Markets Performance

The global stock markets have been reported for their record decline. On 23 March 2020 the S&P 500 Index witnessed an usual drop of 35% compared to the record high on 18 February 2020. In no time at all, the intensity of this record fall became comparable with the financial crisis of 2008, black Monday of 1987, and the great depression of October-November, 1929 (Helppie McFall, 2011). Fernandes (2020) also explained that the US S&P 500 index went down to 30% during March 2020. He further described that the UK and Germany's stock markets were noticed for their worst performance than the US market. The returns of these two markets were fallen by 37 and 33%, respectively. However, the worst performers in the global stock markets were Brazil (−48%) and Columbia (−47%).

Japan's market index dropped more than 20% compared to the record high values of December 2019. S&P 500 Index and Dow Jones share points were declined by 20% in March 2020. The Nikkei Index also reported the same downfall. The Colombo Stock Exchange witnessed a 9% drop in share value and experienced three market halts during mid-March 2020. The Indonesian stock market followed a similar decline. In April 2020, the index was opened with a 64.06 points decline. The UK-FTSE index plunged by 29.72%. The DAX (Germany) index was dropped by 33.37%, CAC (France) by 33.63%, NIKKEI (Japan) by 26.85%, and SUNSEX (India) want down by 17.74% (Machmuddah et al., 2020). Shanghai Composite went down to 2,660.17 points on 23rd March 2020, showing a decline of 12.49% compared to December 2019. KOSPI touched the peak level of 2,204.21 points on 27th December 2019 and dropped to the lowest point of 1,457.64 on 19th March 2020, showing a drop of 33.87%. The BSE SENSEX reported the highest points of 41,681.54 on 20th December 2019. BSE SENSEX plunged to 25,981 points on 23rd March 2020 due to the COVID-19 outbreak, demonstrating a decline of 37.66%. FTSE 100 showed an upward trend on 27th December 2019 with a record index of 7,644.90 points, but it reflected the downward trend followed by a pandemic with an index value of 4,993.89 34.67% decline. The NASDAQ 100 Index reached 8,778.31 points on 26th December 2019 and observed the negative effects of the COVID outbreak by touching 7006.92 points with a declining trend of 20.17%. Moreover, MOEX revealed a bullish trend on 27th December 2019 with an index value of 3,050.47 points and reflected the effects of COVID-19 by reaching 2,112.64 points with the corresponding decline of 30.74%.

Besides, FTSE MIB reached the record level of 24,003.64 points on 20th December 2019 and then touched 14,894.44 points due to pandemic on 12th March 2020 with a declining rate of 37.94%. Nikkei 225 demonstrated an upward trend with the peak value of 24,066.12 on 17th December 2019 and represented the lowest range of 16,552.83 points following the pandemic on 19th March 2020 with the corresponding decline of 31.21%. CAC 40 represented 6,037.39 points on 27th December 2019, consequently faced the sharp jerk of 37.80% on 18th March 2020. DAX exhibited an ascending trend on 16th December 2020 with a peak value of 13,407.66, with the corresponding decline of 8,441.71 on 18th March 2020, signifying an increase of 37.04%. Moving forward, S&P/TSX jumped to 17,180.15 on 24th December 2019 and showed the devastating effects of COVID-19 with the sharp decline of 34.64% on 23rd March 2020. Besides, FTS/JSE reflected 3,513.21 points on 20th November 2020 and affected by the outbreak with a decline of 36.37% on 23rd March 2020 (Investopedia).

However, the global stock markets regained and demonstrated a bullish trend during the days of April 2020. The S&P 500 index increased by 29% and regained the strong position it had held in August 2019 (Cox et al., 2020). Shanghai Composite index further increased by 8.22% in May 2020. KOSPI index showed a bullish trend and the index increased by 27.05%. Similarly, BSE SENSEX recaptured its position and touched 33,717.62 points on 30th April 2020, representing the rise of 22.94%. FTSE 100 secured an 18.33% increase, and the index targeted 6,115.25 points on 29th April 2020. NASDAQ 100 touched 9,485.02 points on 20th May 2020 with the respective rise of 26.12%. MOEX showed an upward trend with a 74.64% increase on 13th April 2020. Also, BOVESPA regained by 23.56% on 29th April 2020 and touched 83.170.80 points. FTSE MIB upbeat and reached 18,067.29 on 29th April 2020. On 20th May 2020, NIKKEI Index climbed at 20,595.15 points, reflecting an increase of 19.62%. Moreover, CAC 40 revived by 19.61% on 29th April 2020. DAX invigorated with the 24.79% increase on 20th May 2020. S&P/TSX touched 15,228 points on 29th April 2020, FTSE/JSE recovered by 27.09% on 20th May 2020, beating the outbreak's negative effect (Investopedia).

The stock market indices worldwide have been categorized in terms of Major Stock Indices, Global Stock Indices, and World Stock Indices etc. The Major World Stock market indices as well as their respective countries have been presented in the Table 1.

**Table 1:** Major world market indices.

**Serial #**	**Index name**	**Country**
1	Dow jones	United States
2	S&P 500	United States
3	Nasdaq	United States
4	Small cap 2,000	United States
5	S&P 500 VIX	United States
6	S&P/TSX	Canada
7	Bovespa	Brazil
8	S&P/BMV IPC	Mexican
9	DAX	Germany
10	FTSE 100	Europe
11	CAC 40	France
12	Euro stoxx 50	Europe
13	AEX	Netherlands
14	IBEX 35	Spain
15	FTSE MIB	Italy
16	SMI	Switzerland
17	PSI 20	Portugal
18	BEL 20	Belgium
19	ATX	Austria
20	OMXS30	Stockholm
21	OMXC25	Copenhagen
22	MOEX	Russia
23	RTSI	Russia
24	WIG20	Poland
25	Budapest SE	Hungary
26	BIST 100	Turkey
27	TA 35	Israel
28	Tadawul all share	Saudi Arabia
29	Nikkei 225	Japan
30	S&P/ASX 200	Australia
31	DJ New Zealand	New Zealand
32	Shanghai	China
33	SZSE component	China
34	China A50	China
35	DJ Shanghai	China
36	Hang Seng	Hong Kong
37	Taiwan weighted	Taiwan
38	SET	Thailand
39	KOSPI	South Korea
40	IDX composite	Indonesia
41	Nifty 50	India
42	BSE sensex	India
43	PSEi composite	Philippines
44	Karachi 100	Pakistan
45	HNX 30	Vietnam

*Source: Investing.com*.

### Graphical Representation of Some Leading Indices

Source of all figures: tradingeconomics.com.

**Figure 1:**
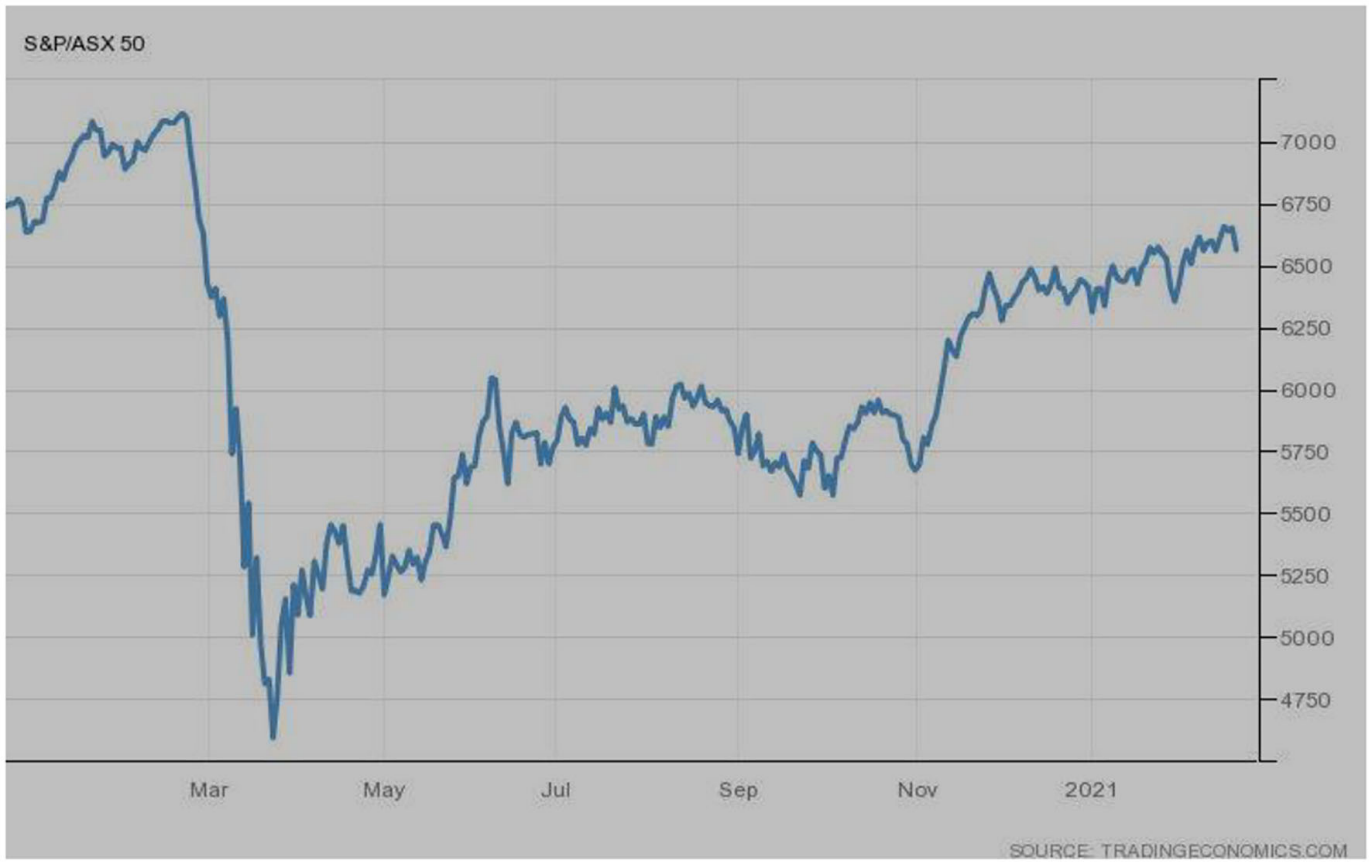
S&P ASX 50 (Australia). Source: tradingeconomics.com. Reproduced with permission.

Figure 1 represents the stock market performance of the S&P ASX 50 index of Australia. It can be seen that the index was performing well-during January 2020, when COVID-19 was at its initial phase. However, March seemed to be a nightmare, when the index plunged and reached the lowest level as COVID-19 spread rapidly and hit a majority of the nations. But the index revived during April 2020, and a gradually limited bullish trend was observed. In-spite of such revival, the index could not reach its peak as the world is still facing the 3rd wave of the pandemic.

**Figure 2:**
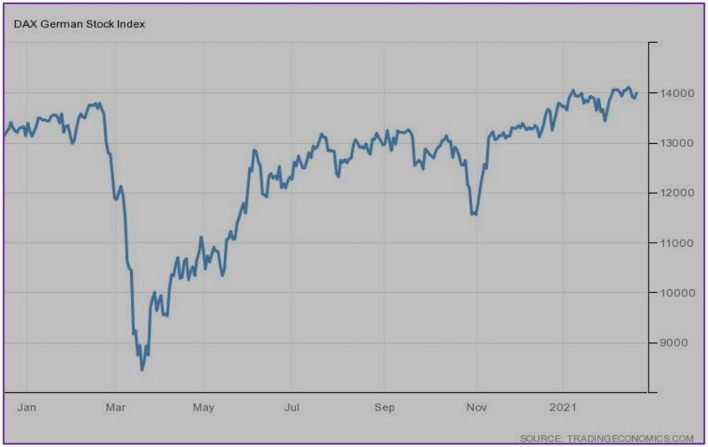
DAX (Germany). Source: tradingeconomics.com. Reproduced with permission.

Figure 2 exhibits the stock market conditions of DAX Germany. The stock market did perform well-until February 2020, it showed a bearish trend in March 2020, followed by a gradual increase, and finally, it realized the position as it was before the pandemic.

**Figure 3:**
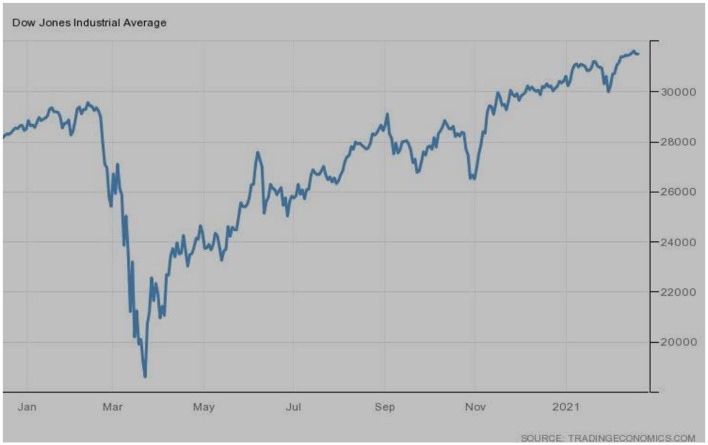
Dow Jones industrial averages (USA). Source: tradingeconomics.com. Reproduced with permission.

Figure 3 demonstrates the stock market situation of Dow Jones Industrial Averages, one of the USA's leading indices. The same situation was observed just like previous indices. The bullish trend was observed before March 2020, followed by the bearish trend during March-April, 2020. Index regained slowly, and revival leads to the extreme upward movements.

**Figure 4:**
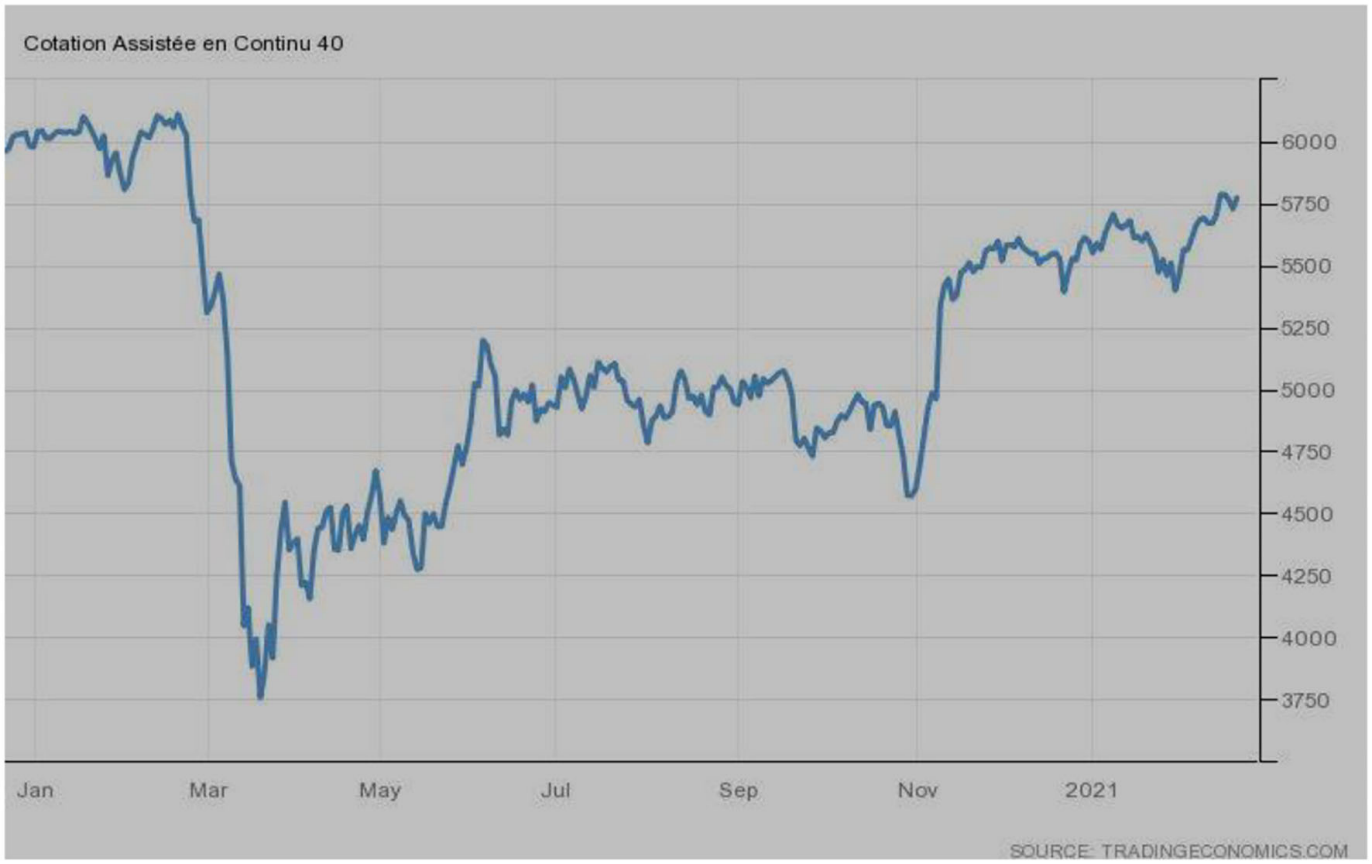
CAC40 (France). Source: tradingeconomics.com. Reproduced with permission.

Figure 4 illustrates the stock market trend of CAC 40, the index of France. The index was at its peak during February 2020. However, a sudden jerk was observed during March 2020, and the index touched the lowest points. The index recovered quite slowly, and to date, it could not recover its previous position. The fluctuations in the index can still be noticed.

**Figure 5:**
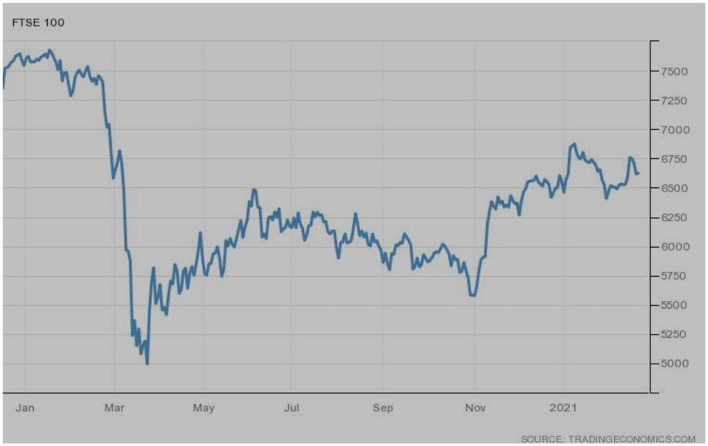
FTSE-100 (Europe). Source: tradingeconomics.com. Reproduced with permission.

The variations in the FTSE 100 index of Europe's market conditions can be seen in Figure 5. The bullish trend can be observed before March 2020, followed by the extreme bearish trend. The index went to the historical lowest points during March 2020. The upward movements were started during April 2020; however, slow movements were there, and the index is still in a slow recovery phase.

**Figure 6:**
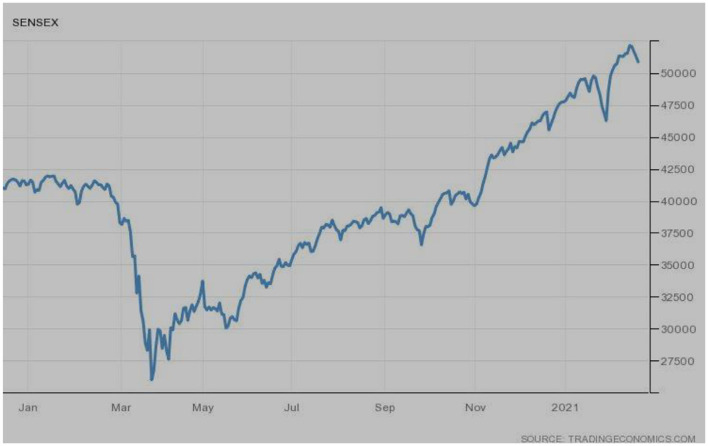
SENSEX (India). Source: tradingeconomics.com. Reproduced with permission.

SENSEX index is a famous stock market index of India. Figure 6 is representing its performance. In January 2020, though the index was not performing very well, it faced the effects of COVID-19 during March 2020. The extreme slow revival was observed after March, and the index remains at the same pace. However, the gradual upward trends lead the index to its highest peak in 2021, as shown in the figure.

**Figure 7:**
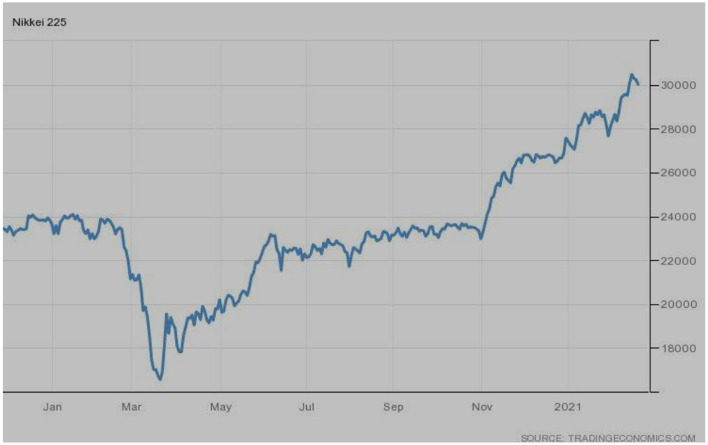
Nikkei-225 (Japan). Source: tradingeconomics.com. Reproduced with permission.

Figure 7 depicts Japan's famous index, i.e., the Nikkei 225. The index was in the recovery phase during January 2020; however, a bearish trend was observed from the outbreak. After March 2020, the recovery phase was there, but static movements were observed. Nevertheless, these slow recoveries finally touched the highest peak in 2021.

**Figure 8:**
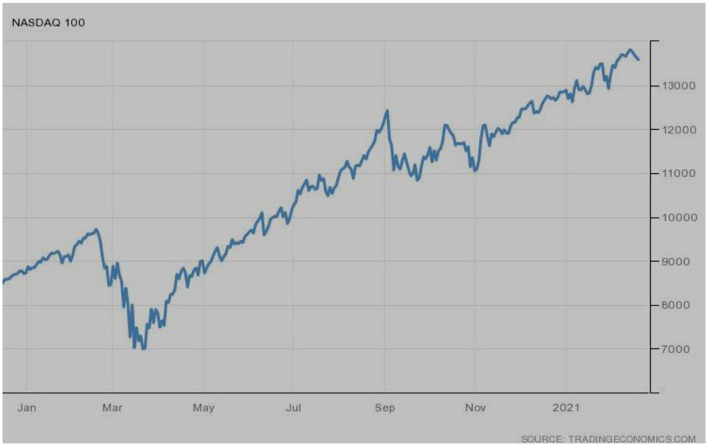
NASDAQ (USA). Source: tradingeconomics.com. Reproduced with permission.

Figure 8 deals with the NASDAQ stock market performance, one of the USA's leading indices. During the start of 2020, its performance was below average, ultimately reaching the lowest points in March 2020 as per the COVID-19 effects. The index escalates gradually, and to date, it jumped and touched the peak level.

**Figure 9:**
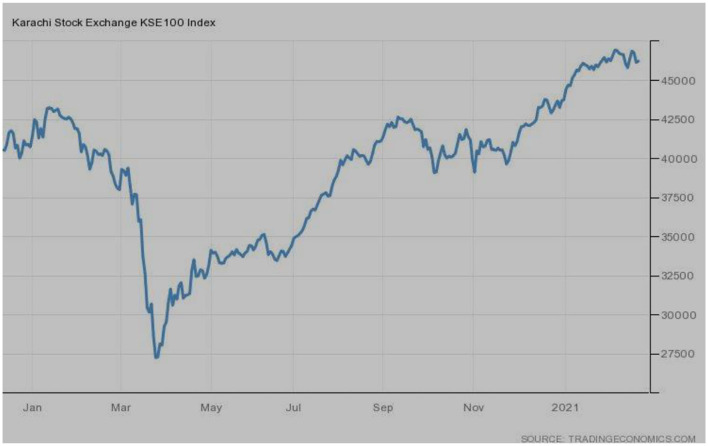
PSX-100 (Pakistan). Source: tradingeconomics.com. Reproduced with permission.

Referring to Figure 9, the PSX-100 of Pakistan was performing well-before the sharp rise of COVID-19 in Pakistan. However, the month of March 2020 proved to be a terrible one; the index plunged and touched the lowest level. A slow revival was observed, which ultimately hit the highest points during 2021, as showing in the Figure 9.

**Figure 10:**
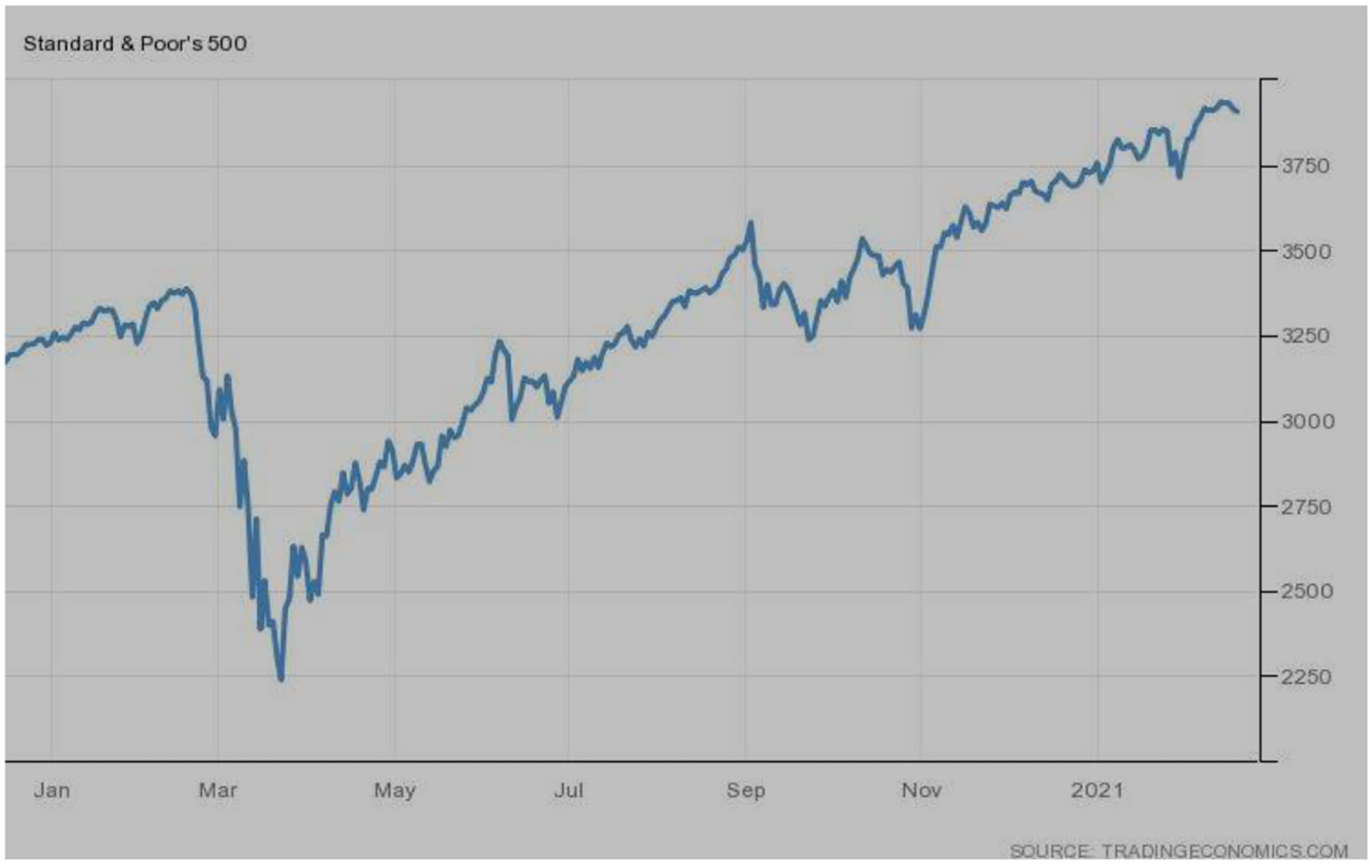
S&P 500 (USA). Source: tradingeconomics.com. Reproduced with permission.

The performance of the S&P 500 index, a prominent index of the USA, seems to be similar to the NASDAQ stock market index. However, before COVID and the recovery phase after March 2020 is better than the NASDAQ index. Currently, the index has reached the highest level as shown in Figure 10.

**Figure 11:**
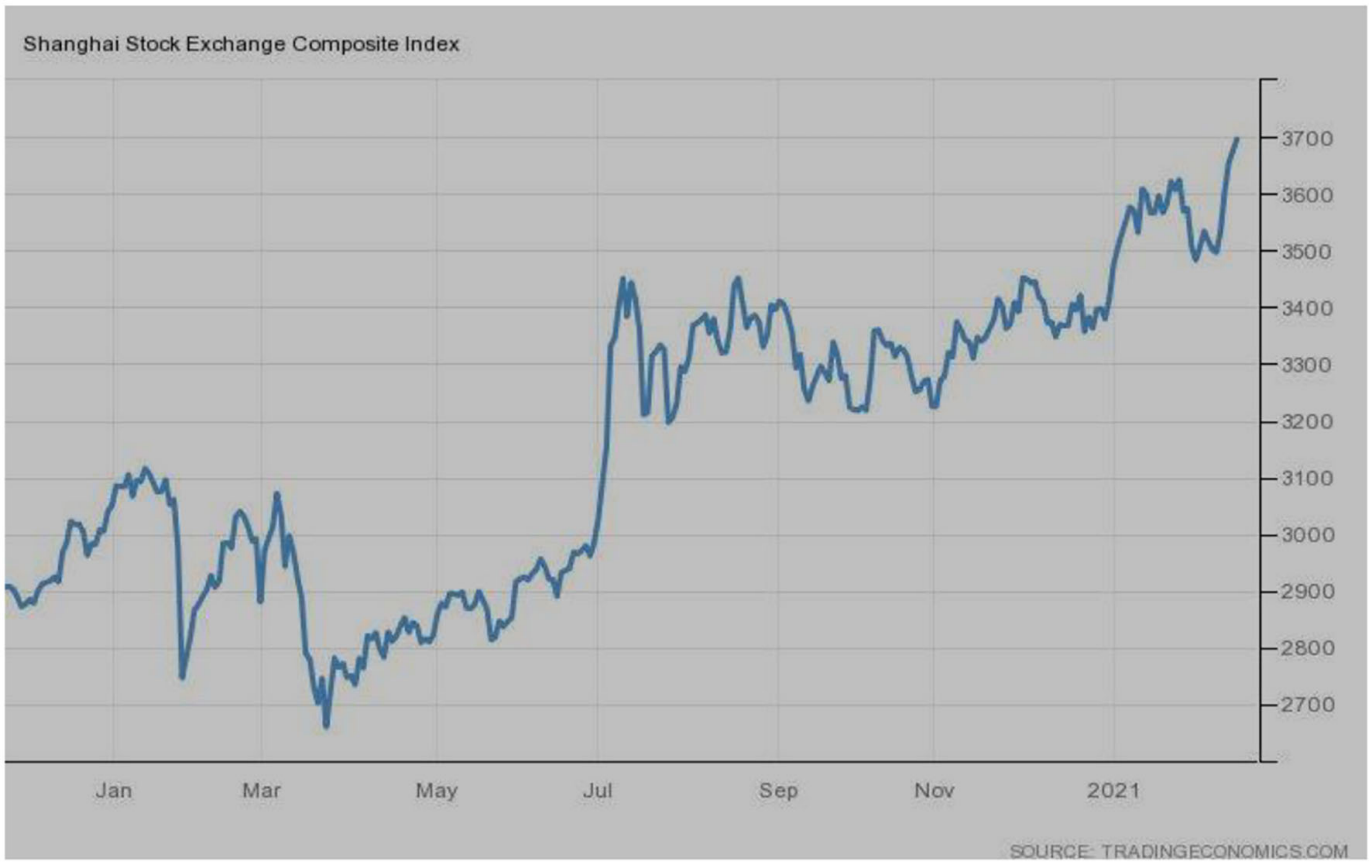
SSE composite (China). Source: tradingeconomics.com. Reproduced with permission.

Figure 11 exhibits the Shanghai Stock Exchange Composite index of China, the origin of the COVID-19 pandemic. The SSE index is the outperformer index of China; however, it was severely affected by the pandemic. The index plunged from the start of the outbreak up to June 2020, followed by a sharp rise and now, with the gradual increase, the index has reached the maximum points.

## Implications for Stock Market Participants

The current study has some implications for market participants and policymakers, i.e., investors, managers, corporations, and governments. The investors must have market know how to invest their resources in favorable avenues even during the contingent market conditions, which have just happened during COVID-19. The investors can take guidance from the mangers and policymakers in this regard. In this way, investors can take rational decision making for their investments. Moreover, investors can generate their portfolios and risk management strategies. Besides, investors must focus on diversification to avoid losses during the pandemic situation.

The managers are the key stakeholders of financial markets; they have experience with stocks' risky nature during the pandemic and can take preventive measures accordingly. The mangers can increase the confidence level of investors, which lead them to make long term investments.

Governments can also play a vital role in assisting with the outbreak in tax rebates and interest-free loans. Governments can even facilitate the national markets by relaxing the lending policies and providing short-term loans on relaxing terms. Governments can conduct surveys and can assist investors in reducing their uncertainty.

Policymakers can develop successful methodologies for balancing financial investments during the outbreak. For this, they can focus on understanding the dynamics of stock markets in devising effective strategies. Besides, policymakers can integrate policies to cope-up with the financial and economic impacts of the COVID-19 outbreak. The emphasis must be on the improvement of stock market stability.

## Unlocking the Future in Post-COVID-19 World

The aim of realizing sustainable growth, a big challenge for all economies, has been underscored by the novel virus. The pandemic has directed that economies are not goal-oriented in terms of their aspiration; they are required to achieve the milestones of a robust global economy. The greatest accomplishments are always achieved through the heights of determination. History is always there to provide lessons for the future. Nearly 75 years ago, amid World War II and one of Britain's most difficult hours, Winston Churchill inspired the whole nation not with the slogans to “reconsider what is achievable” but with a firm determination to “never surrender.” Now at the most difficult time of the century, we are required to continue our fight for what the world needs instead of reconsidering the sustainable development goals. This crisis requires a determined global effort to “build back better” by making a big reset to reach where we were before (Kharas and and McArthur, 2020). Moreover, the leaders of the world must draw a new course of action for improving the functioning of international financial and monetary system to make it strong enough to cope with any such crisis in future (Coulibaly and Prasad, 2020). The stock markets had to face the worst situation in the last 30 years, business operations have abruptly failed, and various economic sectors have been critically affected. However, the best point that came out of the COVID-19 pandemic is the businesses' pressure to be innovative and redefine their operations. One example is the tech community, which has been progressing to facilitate the community in adopting the technology to deal with the pandemic's challenges. Such technological innovations assist specific divisions of organizations or even the whole organizations to carry on their operations irrespective of the current contingent situation. Certainly, the world and the multinational business models will face diverse post-virus issues. Following the COVID-19 pandemic, the nations will observe new policies relating to restructuring and operational strategies, i.e., strategic workforce planning including remote staff planning, flexible conventions, workers proficiency, best practices and HR strategies; Crisis response and business continuity planning, risk control strategies and measures; Financial resources to weather future unforeseen events; Cloud-enabled IT infrastructure (and the attendant improved cybersecurity procedures); and the Redundant sourcing of necessities (inventory, materials and individuals), (David, 2020).

The prospects of the stock markets and the economies are based on the availability and accessibility of the vaccine. The optimism about the vaccine has revitalized the investors' appetite regarding hotels, energy firms, and airlines. However, some others have been brutally affected by the pandemic and are forced to sell their respective market shares. Stock markets are largely dealing with the sentiment that tomorrow may be better than today, leading to a fundamental and perhaps enduring sea change. The development of more vaccines would pave the way for more optimism. What this result demonstrates is that while the virus is not yet beaten, it is beatable. That ray of light has lit up stock markets around the world. As usual, some stock market participants are there to look for something else to worry about (Jack, 2020).

## Conclusion

The current study deals with the impact of the COVID-19 pandemic on the global stock markets. It has focused on the contingent effects of previous and current pandemics on the financial markets. It has also elaborated on the impact of the pandemic on diverse pillars of the economy. The pandemic has severely hit the worldwide markets and posited challenges for economists, policymakers, head of states, international financial institutions, regulatory authorities, and health institutions to deal with the long-lasting effects of the outbreak. It has opened our eyes to concentrate our efforts to protect the future health of citizens and the also financial issues. In the current pandemic situation, the stock markets faced the effects of Covid-19, and this back-and-forth is ongoing. The majority of the world stock markets have suffered trillion-dollar losses (Lyócsa et al., 2020). International financial institutions like IMP and World Bank have been forced to reduce their forecasted growth for 2020 and the years to come (Boone et al., 2020). The global stock markets have been reported for their record decline. The month of March 2020 saw an unusual drop in most worldwide indices like the S&P 500 Index, NASDAQ, NIKKEI, SSE composite, CAC-40; DAX etc. However, the global stock markets regained and demonstrated the bullish trend during the days of April 2020. Irrespective of all these cyclical effects of the pandemic, still hopes are there for the sharp rise and speedy improvements in global stock markets' performance. Moreover, these past events have become a key for mankind to get insights for better future planning (Su et al., 2021).

## Behavioral vs. Conventional Finance

The two polar aspects of finance i.e., traditional finance and behavioral finance have also shed light on the psychology of investors during the COVID-19 pandemic. As far as traditional finance is concerned, investors behave rationally. The rational attitude of investors restricts them from imitating the decisions of others. Investors get the basic facts and figures about the stock markets through their own efforts, resultantly the fear of avoiding future losses compel the investors to sell their stocks and the market shows bearish trend (Jabeen and Rizavi, 2021). The same has happened in the world's stock markets during the peak of pandemic. There have been sudden jerks observed around the global stock markets (Jabeen and Farhan, 2020).

On the other hand, behavioral finance is supposed to consist of a set of theories which focus on the irrationality of investors. The viewpoint of irrationality of investors is the foundation of behavioral finance. The irrational aspect of investors forces them to follow the decision of other investors by setting aside their own information. In such context, investors have confidence on the decision of other investors as they feel that others may possess better information skills (Jabeen and Rizavi, 2021). As a result the panic market conditions lead investors to blindly follow the others to protect their investment and market also depicts the bearish trend, the one which has been seen during the COVID-19 outbreak.

This debate has proven that both the traditional finance and behavioral finance have provided the same mechanism during the COVID-19 pandemic, irrespective of the fact that these pillars of finance deal with the opposing behaviors of investors i.e., rational and irrational. In both of the scenarios, the investors have sale their shares and resultantly the bearish trend has been observed.

## Data Availability Statement

The raw data supporting the conclusions of this article will be made available by the authors, without undue reservation.

## Author Contributions

All authors listed have made a substantial, direct and intellectual contribution to the work, and approved it for publication.

## Conflict of Interest

The authors declare that the research was conducted in the absence of any commercial or financial relationships that could be construed as a potential conflict of interest.

## Publisher's Note

All claims expressed in this article are solely those of the authors and do not necessarily represent those of their affiliated organizations, or those of the publisher, the editors and the reviewers. Any product that may be evaluated in this article, or claim that may be made by its manufacturer, is not guaranteed or endorsed by the publisher.
